# Case of Syringomyelia Resulting from a War Wound

**Published:** 1919

**Authors:** 


**Case of Syringomyelia Resulting from a War Wound.** By MEURIOT et LHERMITTE. *Société de Neurologie*, Jan. 10, 1918.

Very little is known about the pathogeny of syringomyelia. Guillain has tried to show authentic cases consecutive to distal (finger) injuries, this occurring a long time after the healing of the wound. He thinks that the origin of the medullar cavities might be a peripheric infection, migrating from the nerves to the cord. The extension of peripheric nerve lesions to the cord was also shown by Dumenil's case. Then, might it not be possible for infected war-wounds to produce more serious spinal lesions, and particulary syringomyelia? The following observation is of medico-legal interest. F... 34 years old, no pathologic antecedents, was shot in the left thumb in January, 1915. A felon appeared subsequently, causing the exfoliation of the last phalanx. He went back to the trenches and was sent to a depot as instructor in June, 1916. In February, 1917, he noticed that he had difficulty in certain movements of left hand and arm, and that the hand was getting thinner. This atrophy produced deformity of the hand which caused him to be sent to the Neurologic Center in December, 1917, where syringomyelia was found to be quite in evidence. Briefly this man, without any morbid antecedents, many months after an infection of the thumb caused by gun shot, develops progressive symptoms: motor, sensitive, trophic, reflex, vasomotor and oculopupillary, limited to the left side (same side as the wound). This rich and continuous symptomatology imposes a diagnosis of cervical syringomyelia limited to the left side of the cord, same side as the injury. The fact that the lesion appeared only a long time after the infection also suggests the infection as the cause of the disease. The felon itself did not cause any pain, suggesting the pre-existence of a medullar cavity, of which a fundamental characteristic, analgesia, was revealed by the traumatism. The point is not very clear as to whether the felon was the cause of, or simply aggravated, the syringomyelia.

